# Evaluation of the Cytotoxic Activity and Anti-Migratory Effect of Berberine–Phytantriol Liquid Crystalline Nanoparticle Formulation on Non-Small-Cell Lung Cancer In Vitro

**DOI:** 10.3390/pharmaceutics14061119

**Published:** 2022-05-24

**Authors:** Abdullah M. Alnuqaydan, Abdulmajeed G. Almutary, Mohd Azam, Bikash Manandhar, Geena Hew Suet Yin, Lee Li Yen, Thiagarajan Madheswaran, Keshav Raj Paudel, Philip M. Hansbro, Dinesh Kumar Chellappan, Kamal Dua

**Affiliations:** 1 Department of Medical Biotechnology, College of Applied Medical Sciences, Qassim University, Buraidah 51452, Saudi Arabia; abdulmajeed.almutary@qu.edu.sa; 2 Department of Medical Laboratories, College of Applied Medical Sciences, Qassim University, Buraidah 51452, Saudi Arabia; m.aftab@qu.edu.sa; 3 Discipline of Pharmacy, Graduate School of Health, University of Technology Sydney, Sydney, NSW 2007, Australia; bikash.manandhar@uts.edu.au (B.M.); kamal.dua@uts.edu.au (K.D.); 4 Faculty of Health, Australian Research Centre in Complementary & Integrative Medicine, University of Technology Sydney, Ultimo, NSW 2007, Australia; 5 School of Pharmacy, International Medical University, Kuala Lumpur 57000, Malaysia; geena.hewsuet@student.imu.edu.my (G.H.S.Y.); lee.liyen@student.imu.edu.my (L.L.Y.); 6 Department of Pharmaceutical Technology, School of Pharmacy, International Medical University, Kuala Lumpur 57000, Malaysia; thiagarajan@imu.edu.my; 7 Centre for Inflammation, Faculty of Science, School of Life Sciences, Centenary Institute and University of Technology Sydney, Sydney, NSW 2007, Australia; keshavraj.paudel@uts.edu.au (K.R.P.); philip.hansbro@uts.edu.au (P.M.H.); 8 Department of Life Sciences, School of Pharmacy, International Medical University, Kuala Lumpur 57000, Malaysia; dinesh_kumar@imu.edu.my; 9 Uttaranchal Institute of Pharmaceutical Sciences, Uttaranchal University, Dehradun 248007, India

**Keywords:** berberine, liquid crystalline nanoparticles, non-small-cell lung cancer, human lung adenocarcinoma A549 cell, proliferation, migration, mRNA expression, protein expression

## Abstract

Non-small-cell lung cancer (NSCLC) is the most common form of lung cancer, which is a leading cause of cancer-related deaths worldwide. Berberine is an isoquinoline alkaloid that is commercially available for use as a supplement for the treatment of diabetes and cardiovascular diseases. However, the therapeutic benefits of berberine are limited by its extremely low bioavailability and toxicity at higher doses. Increasing evidence suggests that the incorporation of drug compounds in liquid crystal nanoparticles provides a new platform for the safe, effective, stable, and controlled delivery of the drug molecules. This study aimed to formulate an optimized formulation of berberine–phytantriol-loaded liquid crystalline nanoparticles (BP-LCNs) and to investigate the in vitro anti-cancer activity in a human lung adenocarcinoma A549 cell line. The BP-LCN formulation possessing optimal characteristics that was used in this study had a favorable particle size and entrapment efficiency rate (75.31%) and a superior drug release profile. The potential mechanism of action of the formulation was determined by measuring the mRNA levels of the tumor-associated genes *PTEN*, *P53*, and *KRT18* and the protein expression levels with a human oncology protein array. BP-LCNs decreased the proliferation, migration, and colony-forming activity of A549 cells in a dose-dependent manner by upregulating the mRNA expression of *PTEN* and *P53* and downregulating the mRNA expression of *KRT18*. Similarly, BP-LCNs also decreased the expression of proteins related to cancer cell proliferation and migration. This study highlights the utility of phytantriol-based LCNs in incorporating drug molecules with low GI absorption and bioavailability to increase their pharmacological effectiveness and potency in NSCLC.

## 1. Introduction

Lung cancer is the most prevalent cancer, with over 2 million new cases reported in 2020 worldwide [1]. It is also the leading cause of cancer-related deaths globally, with 1.79 million reported deaths (18% of all cancer-related deaths) in 2020 [1]. Non-small-cell lung cancer (NSCLC), making up ~85% of all lung cancer cases, remains the most common form of lung cancer [2]. NSCLC is mainly caused by tobacco smoking, while second-hand smoking, exposure to occupational carcinogens, pollution, and genetic predisposition have also been associated with NSCLC [3]. Within NSCLC, the most common subtype adenocarcinoma can occur in both current or former smokers and non-smokers [1]. Surgical resection, radiotherapy, chemotherapy, and immunotherapy are the standard choices for the management of different stages of NSCLC [4,5]. While surgical resection is the standard of care for patients with stages I and II NSCLC, unresectable or metastatic NSCLC at an advanced stage warrants platinum-based chemotherapy [6]. However, the high toxicity profiles and safety issues of these cytotoxic chemotherapeutic agents present the need for the development of a therapeutic regimen that is effective in the treatment of cancer and metastasis with minimal toxicity.

**Figure 1:**
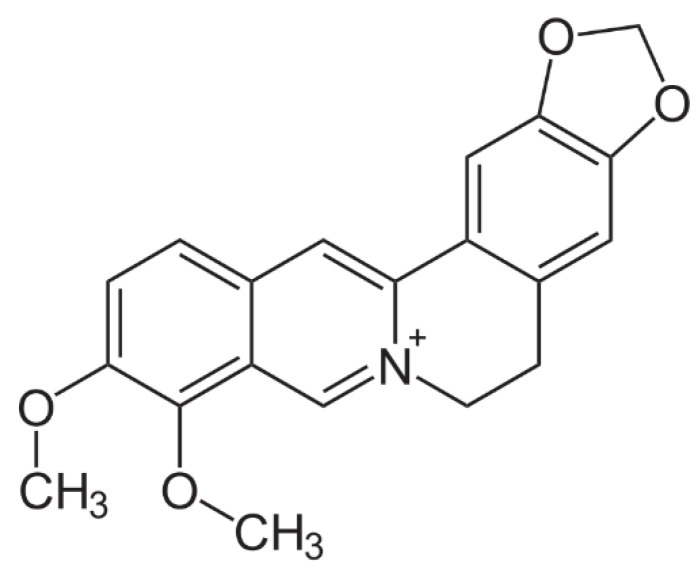
Chemical structure of berberine.

Traditional medicinal plants have provided many beneficial anti-cancer agents such as paclitaxel, vinca alkaloids, camptothecin, curcumin, and boswellic acid. Berberine (Figure 1), a small molecule isoquinoline alkaloid, is present in the roots, rhizomes, and stem bark of several medicinal plants, including *Berberis vulgaris*, *Coptis chinensis*, and *Hydrastis canadensis*, which are traditionally used for the treatment of diarrhea [7,8]. Berberine has been extensively studied for its medicinal properties and has been shown to possess anti-microbial [9,10,11,12], anti-inflammatory [13,14,15], and anti-oxidative properties [16,17,18]. Emerging evidence indicates that berberine is therapeutically effective against various cancers, including lung cancer [19], breast cancer [20], liver cancer [21], prostate cancer [22], cervical cancer [23], and leukemia [14]. Berberine inhibits NSCLC cell proliferation, metastasis, and colony formation in vitro; inhibits the subcutaneously transplanted NSCLC tumor growth; and prolongs the survival of the tumor-bearing mice [24,25]. Berberine, in combination with gefitinib, is currently under phase II clinical trial for the treatment of advanced NSCLC (NCT03486496).

Berberine exerts anti-cancer activity by causing cell cycle arrest and autophagy, promoting apoptosis, and inhibiting angiogenesis of tumor cells [25,26,27,28]. Berberine also suppresses endothelial–mesenchymal transition ability and downregulates the expression of metastasis-related proteins such as matrix metalloproteinase and signaling pathways [26,29]. Berberine selectively inhibits the growth and proliferation of gefitinib-resistant NSCLC cells by causing mitochondrial dysfunction and inhibiting the reactive oxygen species–AMPK–cellular lipogenesis signaling pathway [30]. The berberine-induced inhibition of proliferation and apoptosis has been associated with the upregulation of tumor suppressor protein P53 in vitro and tumor xenograft in mice [31]. *P53* is one of the key tumor suppressor genes and is associated with the regulation of cell division, cell cycle, and apoptosis of cells [32]. Another tumor suppressor gene *PTEN* (encoding phosphatase and tensin homology), which is involved in the regulation of cellular autophagy, is also upregulated by berberine in several models of cancer [33,34]. However, berberine has downsides in terms of its poor lipid solubility, poor gastrointestinal absorption, and low bioavailability, which restricts the application of berberine for therapeutic benefits [35,36]. The nanotechnological approach in formulating and delivering berberine may unlock new possibilities for developing berberine as a therapeutic dosage regimen for the treatment of NSCLCs.

The nanoparticle-based drug carrier system is one of the emerging drug delivery systems for the safe, effective, and controlled delivery of chemotherapeutic agents [37,38,39,40,41]. These nanocarriers have shown promising results in the delivery of potential anti-cancer agents, for example curcumin and boswellic acid, with increased bioavailability and efficacy and decreased toxicity profiles [42,43,44,45,46]. Among the nanoparticle-based formulations, liquid crystalline nanoparticles (LCNs) have been gaining interest as nano-carrier systems for anti-cancer and anti-inflammatory drugs, owing to their potential to improve the bioavailability and stability of the incorporated drug [47]. Monoolein-based LCNs incorporating rutin, naringenin, and berberine have already shown effectiveness in terms of their anti-cancer activity against NSCLC in vitro [48,49,50,51]. However, the anti-cancer potential of berberine-incorporated phytantriol-based LCNs has not yet been investigated.

**Figure 2:**
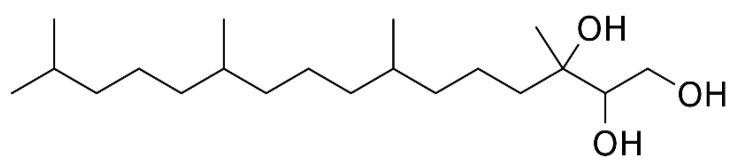
Structure of phytantriol.

**Figure 3:**
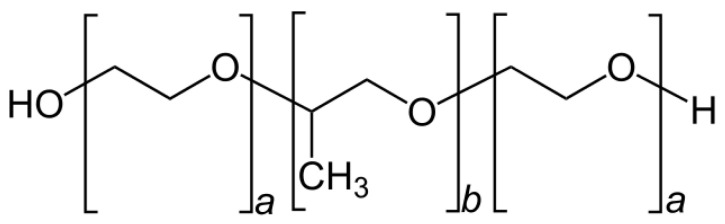
General structure of poloxamer (where poloxamer 407 has block lengths of *a* = 101 and *b* = 56).

Phytantriol (Figure 2) is an amphiphilic lipid commonly used in the production of self-assembled lyotropic LCNs due to its biocompatibility and propensity to form non-lamellar phases in aqueous environments, including inverse bi-continuous cubic and inverse hexagonal phases [52,53]. Recently, the use of phytantriol has been popularized due to its lack of ester and unsaturated bonds, which provides greater chemical stability against hydrolysis and enzymatic degradation [54,55]. A phytantriol-based cubic phase precursor solution was incorporated with the anti-cancer drug hydroxycamptothecine (HCPT), where the results showed that a phytantriol-based cubic phase embolic gelling solution can be a promising carrier for HCPT delivery and sustained drug release by vascular embolization. Incorporating berberine into phytantriol-based LCNs could potentially rectify the solubility and stability issues of berberine. In addition, poloxamer 407 (Figure 3) is a hydrophilic non-ionic surfactant that is employed in the formulation of nanostructures.

The high mortality rate associated with NSCLC is due to the high metastatic nature of NSCLC, which is present in most patients by the time it is diagnosed. Recently, keratin 18 (KRT18), a cytoskeletal protein that is upregulated in most types of tumors, has been associated with the clinical stage, cancer progression, deep tumor invasion, metastasis, and poor survival in NSCLC patients [56]. It was suggested that the knockdown of KRT18 decreases metastasis and increases chemosensitivity. The uncontrolled proliferation and migration of cancer cells are governed by a range of protein signaling pathways [57]. The protein that induces tumor cell proliferation and/or metastasis are usually overexpressed in lung cancer cells as compared to normal cells. Among these proteins, AXL receptor tyrosine kinase (AXL), carbonic anhydrase IX (CA9), enolase 2 (ENO2), human epidermal growth factor receptor (HER) 1, HER2, HER3, progranulin (PRGN), and platelet-derived growth factor-AA (PDGF-AA) are primarily involved in cancer cell proliferation [58,59,60,61,62,63,64,65]. Similarly, proteins such as Dickkopf Wnt signaling pathway inhibitor 1 (DKK1), cathepsin B (CTSB), cathepsin D (CTSD), bcl-2 like protein (BCLX), colony stimulating factor 1 (CSF1), and capping actin protein (CAPG) are involved in cell metastasis, migration, and invasion [66,67,68,69,70,71,72].

This study aimed to formulate and characterize berberine-loaded phytantriol-based LCNs (BP-LCNs) and investigate their anti-cancer potential using adenocarcinomic human A549 alveolar-basal epithelial cells. A549 cells are a commonly used cell line as an in vitro model for NSCLC. The anti-cancer potential of BP-LCNs was assessed by measuring the proliferation, colony formation, and migration of A549 cells. In addition, the mechanism underlying the anti-cancer activity of BP-LCNs was analyzed by determining the regulatory role of BP-LCNs on *KRT18*, *PTEN*, and *P53* mRNA levels and the expression of proteins associated with the proliferation and cell migration or cancer metastasis. This study provides evidence of the promising anti-cancer effects of BP-LCN formulations and a future direction towards the further study of berberine-loaded LCN formulations for the safe and effective management of NSCLCs.

## 2. Materials and Methods

### 2.1. Preparation of BP-LCN Formulation

BP-LCNs were formulated via ultrasonication as described by Paudel et al. [51], with slight modification. The modification in the method involved the solubilization of berberine hydrochloride (Merck, Kenilworth, NJ, USA) in phytantriol (Tokyo Chemical Industry, Tokyo, Japan), instead of monoolein, followed by dispersion in Poloxamer 407 (P407, Merck) surfactant solution to obtain the BP-LCN formulation. Briefly, 120 mg of phytantriol was weighed in a glass vial. In a separate glass vial, 12 mg of Poloxamer 407 was dissolved in 5 mL of distilled water to prepare the surfactant solution. Both vials were heated at 70 °C in a water bath to attain equilibrium. Then, 5 mg of berberine was weighed and solubilized in the molten phytantriol. Then, the mixture was immersed into a sonicator bath for 5 min at 100 Hz to achieve complete dissolution. To this mixture, the P407 solution was added to form a coarse dispersion. Finally, the coarse dispersion was size-reduced using a probe sonicator (Labsonic P, Sartorius, Göttingen, Germany), maintained at an amplitude of 60 with repeated cycles of turning on (for 5 s) and off (for 5 s) over a period of 15 min. Blank LCNs were prepared using the same method without adding berberine.

### 2.2. Optimization of Formulation and Process Parameters

Central composite design (CCD), a type of response surface methodology (RSM), was utilized for statistical optimization and modeling of critical factor effects on selected critical quality attributes (CQA) of the product. Design-Expert version 12 (v12) software was used to generate regression equations, contour plots, and response surface plots to visualize the responses to various levels of the independent variables (Table 1). The numerical optimization function was used to target a particle size (PS) of 200 nm and to maximize the entrapment efficiency (EE). A total of nine formulations (BP1-BP9) were prepared as per the three-level factorial design (Table 1).

### 2.3. Physicochemical Characterization of Formulation

The particle size, polydispersity index (PDI), and zeta potential values of the BP-LCNs were measured using a Zetasizer Nano ZS (Malvern Instruments, Malvern, UK) with a laser beam wavelength of 632.8 nm at a fixed angle of 90°. The samples were diluted (dilution: 1:50) with distilled water prior to the analysis. All data were measured in triplicate at 25 °C.

### 2.4. Entrapment Efficiency (EE)

The EE was estimated via ultrafiltration using a specialized Amicon ultra-4 ultrafilter tube (MWCO 10,000 g/mol, Merck). A total volume of 1 mL of the BP-LCN dispersion was transferred to the upper chamber of an ultrafilter tube and centrifuged via a high-speed centrifuge (Eppendorf centrifuge 5810R, Eppendorf, Germany) at 2500× *g* for 15 min at 25 °C. The filtrate and lysed dispersion were evaluated using a UV–Vis spectrophotometer (UV-1800, Shimadzu AS, Kyoto, Japan) at a wavelength of 349 nm to determine the free drug (D_free_) and total drug (D_total_) concentrations. The EE was calculated using the following equation:

$$\%EE=\frac{D_{total}-D_{free}}{D_{total}}\times 100$$

where *EE* represents the entrapment efficiency, *D_total_* represents the total amount of drug in the formulation, and *D_free_* represents the total amount of free drug in the formulation.

### 2.5. Morphology

One drop of diluted BP-LCNs (dilution: 1:20) was loaded onto a carbon-coated copper grid. This was allowed to air-dry prior to inspection. The surface morphology of BP-LCNs was analyzed using a transmission electron microscope (Fischione Instrument Inc., Export, PA, USA).

### 2.6. In Vitro Release Study

The dialysis bag method was employed to determine the in vitro release of the drug from the BP-LCN formulation. Dialysis bags (SpectraPor, MWCO 14,000 g/mol, Merck) were filled with 1 mL of BP-LCN dispersion, clamped, and submerged into 30 mL of release medium (phosphate buffer, pH 7.4 ± 0.2) in 50 mL conical tubes (Corning, Corning, NY, USA). The tubes were placed into a shaking water bath at a constant temperature of 37 °C (SW22 Julabo, shaken at 50 strokes/min). Then, 1 mL samples were withdrawn from the medium at fixed time intervals of 0.5, 1, 2, 4, 6, 8, 10, 12, 24, 40, and 72 h, whereby an equivalent volume of fresh medium was replaced to maintain a constant volume. The amount of berberine in the samples was quantified by measuring the absorbance with a UV–Vis spectrophotometry at a wavelength of 349 nm.

### 2.7. Cell Culture

A549 human lung epithelial carcinoma cells (ATCC, Manassas, VA, USA), provided as a kind gift by Prof. Alaina Ammit, Woolcock Institute of Medical Research, Sydney, were grown in low-glucose Dulbecco’s modified Eagle’s medium (DMEM, Lonza, Basel, Switzerland) culture medium (DMEM supplemented with 5% (*v*/*v*) fetal bovine serum (FBS, Lonza) and 1% (*v*/*v*) penicillin and streptomycin (Lonza)) in an incubator, maintained at 37 °C and 5% CO_2_ in a humidified condition. All cell incubations involved in this study were performed in DMEM cell culture medium in the absence or presence of BP-LCNs at 37 °C and 5% CO_2_, unless stated otherwise.

### 2.8. Measurement of Cell Proliferation

#### 2.8.1. MTT Assay

The proliferation of A549 cells in the absence or presence of BP-LCNs was assessed via MTT (3-(4,5-dimethylthiazol-2-yl)-2,5-diphenyl tetrazolium bromide, Merck) assay, as described by Paudel et al. [73,74]. Briefly, A549 cells were seeded in a clear-bottom, transparent 96-well plate (Corning) and grown until 80% confluent. The cells were incubated at 37 °C for 24 h in the absence or presence of BP-LCNs (final concentration of 0.5, 1, 2.5, or 5 µM). The cells were then incubated at 37 °C for a further 4 h in the presence of MTT (dissolved in PBS, final concentration 250 µg/mL). The supernatant was removed, and the formazan crystals were dissolved with 100 µL of dimethyl sulfoxide (Merck) per well. The absorbance was measured using a POLARstar Omega microplate reader (BMG Labtech, Ortenberg, Germany) at an emission wavelength of 540 nm. The inhibitory effect of BP-LCNs on the cell proliferation rate was assessed by calculating the percentage of viable cells that were treated with BP-LCNs relative to the cells that were not treated with BP-LCNs.

#### 2.8.2. Trypan Blue Staining

A549 cells were seeded at a density of 20,000/well in a 48-well plate (Corning). After 80% confluency, the cells were incubated at 37 °C for 24 h in the absence or presence of BP-LCNs (final concentration of 0.5, 1, 2.5, or 5 µM). The cells were washed 1× with PBS, harvested with trypsin, and centrifuged at 300× *g* for 4 min. The supernatant was removed, and the cell pellets were resuspended with PBS. Next, 10 µL of cell suspension was mixed with trypan blue solution (0.4% (*w*/*v*), Thermo Fisher Scientific, Waltham, MA, USA) at a ratio of 1:1 and the number of viable cells was counted under a phase-contrast microscope using a hemacytometer (Hawksley and Sons Ltd., Lancing, UK) as previously described [49].

### 2.9. Wound Healing Assay

The effect of BP-LCNs on the cell migration of A549 cells was assessed via wound healing assay, as previously described [75]. Briefly, A549 cells were seeded at a density of 3 × 10^5^/well in 6-well plates and grown until 80% confluency. A wound was created by scratching the cell monolayer with the tip of a sterile pipette. The detached cells were removed by washing the cells once with PBS. The cells were then incubated at 37 °C for 24 h in the absence or presence of BP-LCNs (final concentration 1, 2.5, or 5 µM). Images were taken using a phase contrast microscope with an objective lens of 10× magnification at 0 and 24 h of incubation with or without BP-LCNs. The width of the wound in the images was calculated. The wound closure was calculated as a percentage (%) of difference in the width of the wound between 0 h and 24 h relative to that at 0 h, as described below.

$$\;Wound\;closure\;\left(\%\right)=\frac{\left(Width\;of\;wound\;at\;0\;h-Width\;of\;wound\;at\;24\;h\right)}{Width\;of\;wound\;at\;0\;h}\times 100\%$$

### 2.10. Colony Formation Assay

A549 cells were seeded at a density of 500/well in 6-well plates. The cells were treated with or without BP-LCNs (final concentration of 0.5, 1, 2.5, or 5 µM) and incubated at 37 °C for 2 weeks. The culture medium with or without BP-LCNs was replaced every 48 h. The cells were washed once with PBS and fixed at room temperature for 20 min with 3.7% (*v*/*v*) formaldehyde. The cells were washed 3× with PBS, then stained with 0.4% crystal violet (Merck). The cells were again washed 3× with PBS. Images of wells were taken from the bottom side of the plates, as previously described [49].

### 2.11. RNA Isolation and Real-Time qPCR

A549 cells, grown on 6-well plates, were incubated at 37 °C for 24 h in the absence or presence of BP-LCNs (final concentration 5 µM). Total RNA was isolated as described previously [76,77]. Briefly, the cells were washed 2× with PBS, then lysed with 400 µL of TRI reagent (Merck) and collected in 1.5 mL Eppendorf tubes. Here, 250 µL of chloroform was added to each tube and the tubes were pulse-vortexed for 5 s. The tubes were incubated at room temperature for 10 min, then centrifuged at 4 °C (12,000× *g* for 15 min). The aqueous phase was transferred into fresh Eppendorf tubes and RNA was precipitated by adding 500 µL of ice-cold isopropyl alcohol. The tubes were pulse-vortexed (for 5 s) and incubated at room temperature for 10 min. The tubes were pulse-vortexed again and centrifuged (12,000× *g*, 10 min, room temperature). The supernatant was removed, then RNA pellets were dislodged and washed with 1 mL of 75% ethanol. The tubes were then centrifuged again (8000× *g*, 5 min, 4 °C). The RNA pellets were further washed and centrifuged at 8000× *g*, 5 min, 4 °C. Ethanol was removed and the RNA pellets were left to air-dry for 15 min on ice. They were then resuspended with nuclease-free water. The concentration and purity of RNA were determined using a Nanodrop One instrument (Thermo Fisher Scientific).

The cDNA was prepared from 1000 ng of RNA following DNase treatment with a DNase I kit (Merck). Random primers (500 ng/µL), dNTPs (10 mM), MMLV reaction buffer (1×) and DTT (100 mM) were added to the reaction mix. Reverse transcription was performed via the subsequent steps of denaturation of RNA (65 °C for 15 min), annealing of primers (25 °C for 10 min), and reverse transcription (37 °C for 50 min) and inactivation of enzymes (70 °C for 15 min) using a Mastercycler Nexus GSX1 thermal cycler (Eppendorf, Hamburg, Germany). Real-time qPCR was performed with 25 ng cDNA, iTaq Universal SYBR Green supermix (1×, BioRad, Hercules, CA, USA), and 5 µM of each of the forward and reverse primers using a CFX96 real-time PCR detection system (initial polymerase activation and cDNA denaturation at 95 °C for 30 s, followed by 40 cycles of denaturation at 95 °C for 15 s, annealing and extension at 60 °C for 30 s, and plate reading). The forward and reverse primers for *KRT18* (forward: GGAAGTAAAAGGCCTACAAG; reverse: GTACTTGTCTAGCTCCTCTC), *PTEN* (forward: GGCTAAGTGAAGATGACAATC; reverse: GTTACTCCCTTTTTGTCTCTG), *P53* (forward: ACCTATGGAAACTACTTCCTG; reverse: ACCATTGTTCAATATCGTCC) and *GAPDH* (forward: TCGGAGTCAACGGATTTG; reverse: CAACAATATCCACTTTACCAGAG) were purchased from Merck.

### 2.12. Protein Array

The expression levels of cancer-related proteins in A549 cells in the presence or absence of BP-LCNs were determined using a Human XL oncology array kit (R&D Systems, Minneapolis, MN). A549 cells were incubated for 24 h at 37 °C in the absence or presence of BP-LCNs (final concentration 5 µM). The cells were washed once with ice-cold PBS, lysed with RIPA buffer containing protease and phosphatase inhibitor (Roche Diagnostics, Basel, Switzerland), and stored at −80 °C until use. The protein concentrations in the cell lysates were determined via bicinchoninic acid (BCA) protein assay using a Pierce BCA protein assay kit (Thermo Fisher Scientific) as compared against the standard BSA curve (0−1 mg/mL concentration). Cell lysates (equivalent to 300 µg protein for each sample) were run on a Human XL oncology array following the manufacturer’s protocol. The protein signals obtained in the array were imaged with ChemiDoc MP imaging system (Bio-Rad, Hercules, CA, USA). The pixel densities of the protein signals in the images were quantified using Image J software (version 1.53c, Bethesda, MD, USA).

### 2.13. Statistical Analysis

The values are represented as means ± SEM or means ± SD, as indicated in the legends. Graph Pad prism version 9.3 (San Diego, CA, USA) was used to perform statistical analyses. Statistical comparisons were performed using unpaired, two-tailed Student’s *t* test or one-way ANOVA followed by Dunnett’s test, as appropriate. A value of *p* < 0.05 was considered statistically significant.

## 3. Results

### 3.1. Preparation and Optimization of BP-LCNs

**Figure 4:**
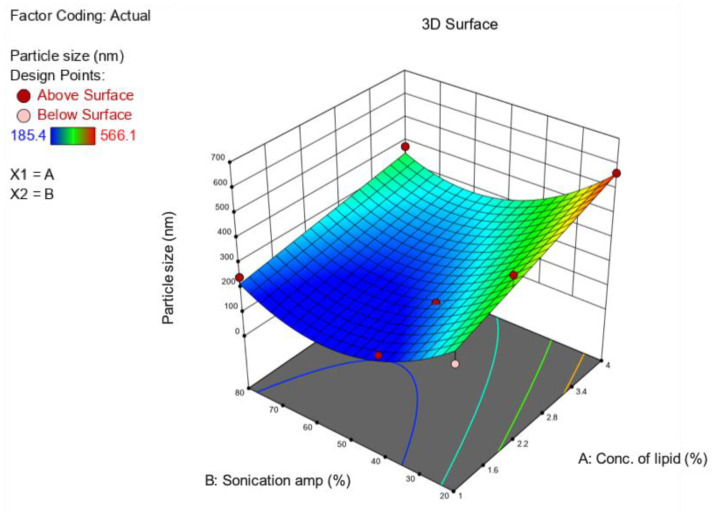
The 3D response surface plot showing the effects of the lipid concentration (**A**) and sonication amplitude (**B**) on the particle size of berberine-loaded phytantriol-based LCNs.

**Figure 5:**
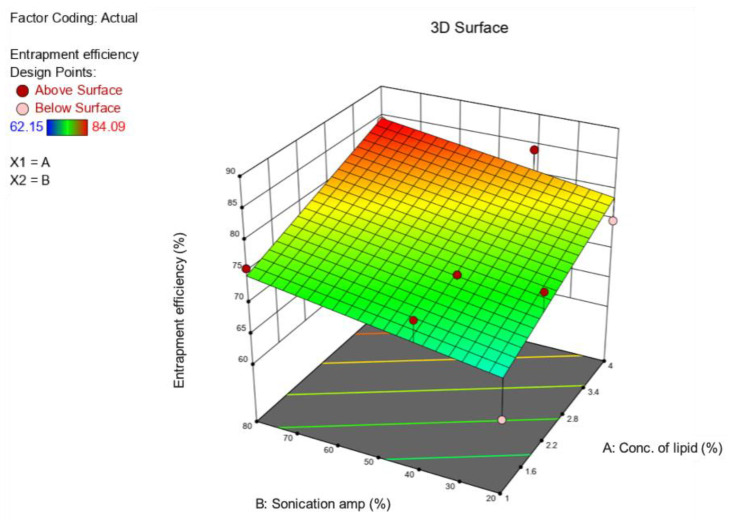
The 3D response surface plot showing the effects of the lipid concentration (**A**) and sonication amplitude (**B**) on the entrapment efficiency of berberine-loaded phytantriol-based LCNs.

The prepared BP-LCNs appeared as a yellowish, cloudy dispersion mixture, whereas the blank LCNs were presented as a white, cloudy dispersion. The formulation parameters used for preparation of BP-LCNs are listed in Table 1. Overall, the results showed PS values with a range of 185 nm to 566 nm and EE values with a range of 62% to 84% (Table 1). The 3D response surface (Figure 4 and Figure 5) and polynomial equations (Table 2 and Table 3) were generated using Design Expert Software version 12 (Minneapolis, MN, USA). The finalized reduced quadratic and linear equations with respect to the variables for PS (Y1) and EE (Y2) are shown in Equations (1) and (2), respectively.

Equation (1):

Y1 = 214.03 + 88.83 A − 82.62 B − 23.44 AB + 0.0525 A² + 153.95 B² (1)

Equation (2):

Y2 = 76.67 + 5.00 A + 2.91 B(2)

In ANOVA, the model F-values of 18.34 and 9.39 for the PS and *EE* models, respectively, implied that both models were significant (*p* < 0.05) (Table 2 and Table 3). Model terms A, B, and B^2^ had significant effects on PS (Table 2), whereas A had a significant effect on *EE* values of the LCNs (Table 3). Factors A and B^2^ of multiple regression Equation (1) showed positive effects on the PS values of BP-LCNs, demonstrating increases in A and B^2^, resulting in larger PS values (Table 2). Factor B, however, showed a negative effect on the PS (Table 2). With regards to the linear model of Equation (2), both factors A and B showed positive effects on *EE* (Table 3). However, factor B was non-significant (*p* > 0.05).

The relationship between the factors and response variables were elucidated using the 3D response surface plots. Increases in lipid concentrations increased the PS values, whereas at high lipid concentrations (>2.8%), increases in sonication amplitudes up to 60% decreased PS to approximately 200 nm, after which PS was increased up to 300 nm (Figure 4).

Increases in lipid concentration increased EE, while at low lipid concentrations (<2.2%) changes in sonication amplitude showed no prominent effects on EE (Figure 5). However, at higher lipid concentrations (>2.2%), increases in sonication amplitude increased EE (Figure 5).

Design Expert^®^ Software version 12 (Minneapolis, MN, USA) was used to generate desirability plots to predict the parameters for an optimized formulation. Thus, a PS of 200 nm and a maximal EE of 77.49% can be achieved at a lipid concentration of 2.4% and sonication amplitude of 60%.

### 3.2. Physicochemical Characterization of Optimized Formulation

**Figure 6:**
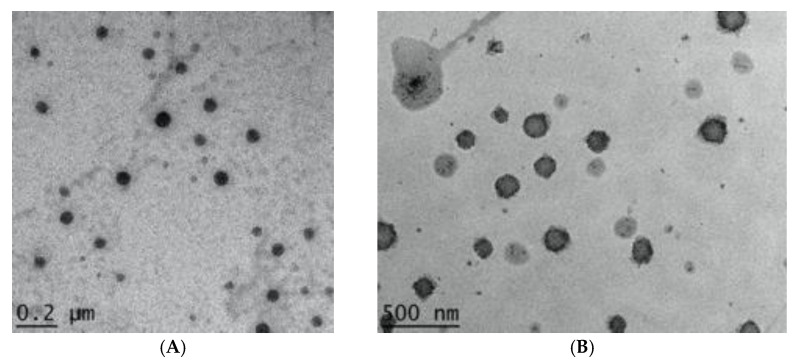
TEM images showing (**A**) blank LCNs and (**B**) optimized BP-LCNs.

The encapsulation efficiency results showed that about 75% of berberine was encapsulated in the BP-LCNs formulation (Table 4). The TEM visualization (Figure 6) revealed that BP-LCNs were small and spherical with a flower-like structure, in addition to being monodispersed and uniform in size (Figure 6B).

**Figure 7:**
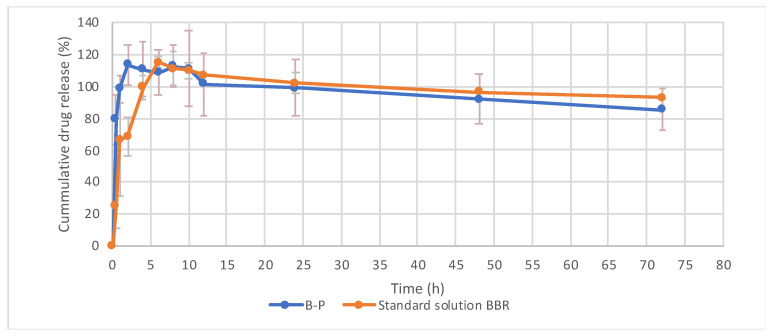
Cumulative release profile of berberine from LCNs. Each value represents mean ± SD (n = 3). B-P refers to berberine–phytantriol nanoformulation; BBR refers to standard berberine.

The in vitro drug release profile of berberine from the BP-LCN formulation was determined at pH 7.3 ± 0.2, 37 °C, with drug released throughout a 72 h period. The initial rapid release from BP-LCNs was observed in the first 2 h (Figure 7). However, the cumulative percentage exceeded 100%, followed by a steady decline (Figure 7).

### 3.3. BP-LCNs Inhibited Proliferation of A549 Cells in a Dose-Dependent Manner

**Figure 8:**
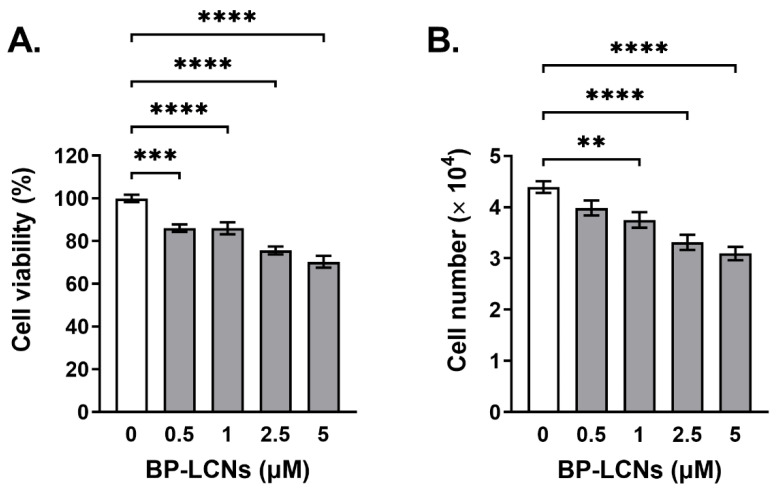
Effect of BP-LCNs on proliferation of A549 cells. A549 cells were incubated at 37 °C for 24 h in the absence or presence of BP-LCNs (final concentration of 0.5, 1, 2.5, or 5 µM). They were then incubated at 37 °C for 4 h with MTT solution (final concentration 250 µg/mL) or stained with trypan blue. Formazan crystals were dissolved with DMSO. and the absorbance was measured by microplate reader (**A**). Trypan blue stained cells were counted using a cell counter (**B**). The values represent Mean ± SEM of 3 independent experiments. Note: ** *p* < 0.01, *** *p* < 0.001, **** *p* < 0.0001. Abbreviation: BP-LCNs, berberine–phytantriol liquid crystalline nanoparticles.

Based on the results of the MTT assay, the incubation of A549 cells with BP-LCNs showed a dose-dependent decrease in the rate of cell proliferation (Figure 8). The proliferation of cells decreased by 14.0% (*p* < 0.001) and 29.7% (*p* < 0.0001) when the cells were incubated with BP-LCNs at final concentrations of 0.5 (lowest concentration used) and 5 µM (highest concentration used), respectively, relative to the cells that were incubated in the absence of BP-LCNs (Figure 8A, proliferation rate: 100 ± 1.7% for cells incubated without BP-LCNs, 86.0 ± 1.8% for cells incubated with 0.5 µM BP-LCNs, and 70.3 ± 2.7% for cells incubated with 5 µM BP-LCNs).

The trypan blue staining and cell count results showed similar dose-dependent decreases in the proliferation of A549 cells when the cells were incubated in the presence of BP-LCNs. The number of viable A549 cells that were not incubated with BP-LCNs was 4.4 ± 0.1 × 10^4^ cells. The inclusion of BP-LCNs in the incubation at final concentrations of 1, 2.5, and 5 µM decreased the number of viable cells by 13.6% (Figure 8B, 3.8 ± 0.2 × 10^4^ cells, *p* < 0.01), 25.0% (Figure 8B, 3.3 ± 0.2 × 10^4^ cells, *p* < 0.0001), and 29.6% (Figure 8B, 3.1 ± 0.1 × 10^4^ cells, *p* < 0.0001), respectively, compared to cells that were not incubated with BP-LCNs.

### 3.4. BP-LCNs Inhibited Migration of A549 Cells in a Dose-Dependent Manner

**Figure 9:**
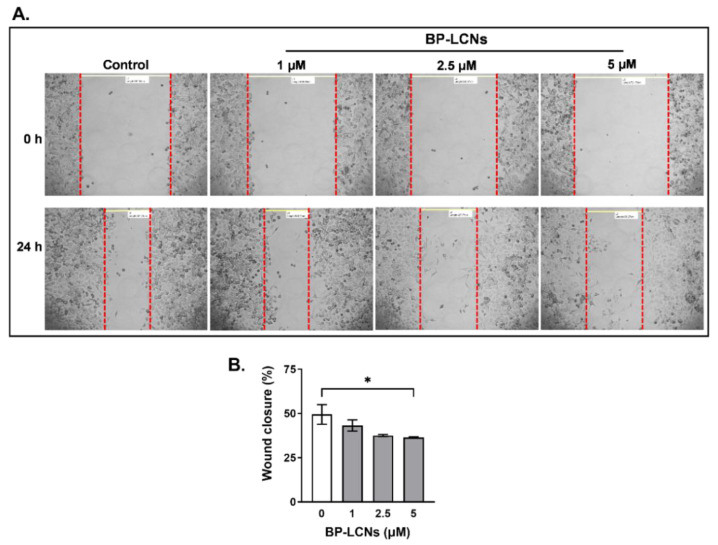
Effect of BP-LCNs on migration of A549 cells. A549 cells were grown in a 6-well plate until confluency. The wound was created by scratching the cell monolayer with the tip of a sterile pipette. The cells were then incubated at 37 °C for 24 h in the absence or presence of BP-LCNs (final concentration 1, 2.5, or 5 µM). Images were taken using a phase contrast microscope with an objective lens of 10× magnification at 0 h and 24 h of incubation with or without BP-LCNs (**A**). The wound closure (%) was quantified by comparing the widths of the wound at 0 h and 24 h for each well (**B**), as explained in the Materials and Methods section. The values in (**B**) represent means ± SEM of 3 independent experiments. Note: * *p* < 0.05. Abbreviation: BP-LCNs, berberine–phytantriol liquid crystalline nanoparticles.

The results from wound healing assay showed that BP-LCNs inhibit the migratory activity of A549 cells in a dose-dependent manner (Figure 9). After 24 h of scratching the wound, the cells that were incubated in the absence of BP-LCNs (control) showed 49.4 ± 5.5% wound closure. The inclusion of BP-LCNs at final concentrations of 1 and 2.5 µM showed decreases in wound closure of 43.2 ± 3.1 and 37.6 ± 0.6%, respectively, relative to the control (Figure 9). However, these differences were not statistically significant. When the final concentration of BP-LCNs in the incubation was increased to 5 µg/mL, the wound closure decreased by 26.3% relative to the control (Figure 9, 49.4 ± 5.5% for control vs. 36.4 ± 0.4% for cells incubated with 5 µM BP-LCNs, *p* < 0.05).

### 3.5. BP-LCNs Inhibited the Colony Formation of A549 Cells in a Dose-Dependent Manner

**Figure 10:**
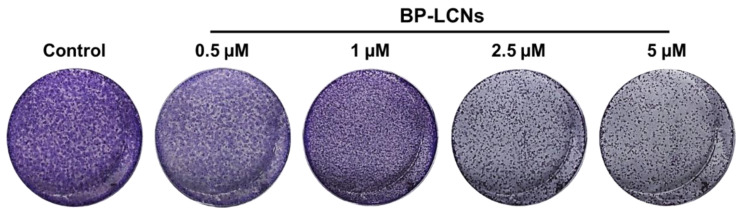
Effects of BP-LCNs on colony formation of A549 cells. A549 cells, grown in a 6-well plate, were incubated at 37 °C for 2 weeks in the absence or presence of BP-LCNs (final concentration of 0.5, 1, 2.5, or 5 µM). They were fixed with formaldehyde, then stained with 0.4% *v*/*v* crystal violet staining solution. Images of individual wells were captured from bottom of the plate. The images are representative of 3 independent experiments. Abbreviation: BP-LCNs, berberine–phytantriol liquid crystalline nanoparticles.

The images of crystal-violet-stained A549 cells that were incubated for 2 weeks with or without BP-LCNs were visually analyzed. The images showed that the inclusion of BP-LCNs in the incubation clearly inhibited the formation of colonies of A549 cells in a dose-dependent manner, relative to the cells that were not incubated with BP-LCNs (control) (Figure 10). The number of colonies, observed with crystal violet staining, were visibly reduced and the gaps between the colonies were increasingly visible with the increase in the final concentration of BP-LCNs in the incubation (Figure 10).

### 3.6. BP-LCNs Decreased the mRNA Levels of KRT18 and Increased mRNA Levels of PTEN and P53

**Figure 11:**
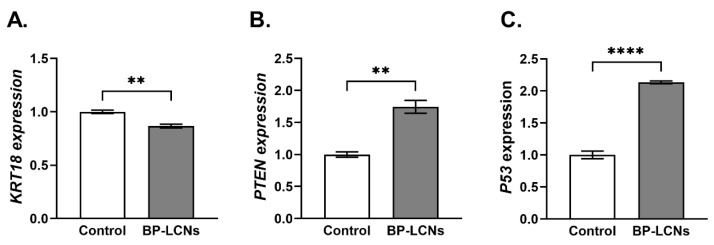
Effects of BP-LCNs on mRNA levels of *KRT18*, *PTEN*, and *P53* in A549 cells. A549 cells were incubated at 37 °C for 24 h in the absence or presence of BP-LCNs (final concentration 5 µM). The figure shows mRNA levels of *KRT18* (**A**), *PTEN* (**B**), and *P53* (**C**). The mRNA levels were measured by RT-qPCR and normalized against *GAPDH*. The values represent Mean ± SEM of 3 independent experiments. Note: ** *p* <0.01, **** *p* <0.0001. Abbreviations: *KRT18*, gene encoding keratin 18; *PTEN*, gene encoding phosphatase and tensin homology; and *P53*, gene encoding tumor protein P53). Abbreviation: BP-LCNs, berberine–phytantriol liquid crystalline nanoparticles.

The incubation of A549 cells with 5 µM concentration of BP-LCNs decreased the mRNA levels of *KRT18* by 13.3% (Figure 11A, *p* < 0.01) compared to the cells that were incubated without BP-LCNs (control). On the other hand, the mRNA levels of *PTEN* and *P53* were increased by 113.3% (Figure 11B, *p* < 0.0001) and 74.7% (Figure 11C, *p* < 0.01), respectively, in the cells that were incubated with 5 µM BP-LCNs relative to control.

### 3.7. BP-LCNs Decreased Expression of Proteins Associated with Proliferation in A549 Cells

**Figure 12:**
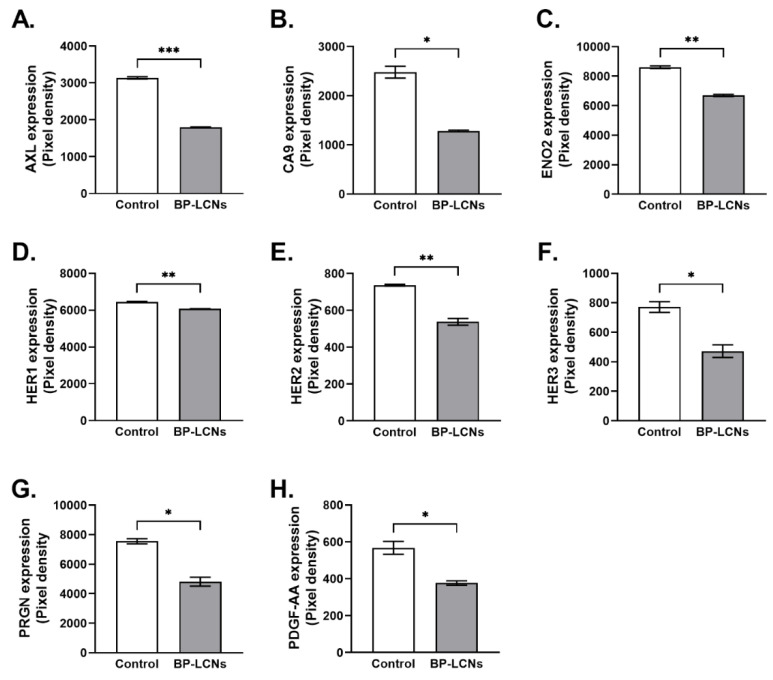
Effects of BP-LCNs on expression levels of proteins related to proliferation in A549 cells. A549 cells were incubated at 37 °C for 24 h in the absence or presence of BP-LCNs (final concentration 5 µM). The figure shows protein expression levels of AXL (**A**), CA9 (**B**), ENO2 (**C**), HER1 (**D**), HER2 (**E**), HER3 (**F**), PRGN (**G**), and PDGF-AA (**H**). The protein expression levels were determined using an oncogene protein array kit and by quantifying the pixel densities of the respective proteins. The values represent means ± SEM of 3 independent experiments. Note: * *p* < 0.05, ** *p* < 0.01, *** *p* < 0.001. Abbreviations: BP-LCNs, berberine–phytantriol liquid crystalline nanoparticles; AXL, AXL receptor tyrosine kinase; CA9, carbonic anhydrase IX; ENO2, enolase 2; HER1, human epidermal growth factor receptor 1; HER2, human epidermal growth factor receptor 2; HER3, human epidermal growth factor receptor 3; PRGN, progranulin; and PDGF-AA, platelet-derived growth factor-AA.

The results from a protein profiler array indicated that the presence of BP-LCNs in the incubation significantly downregulated the expression levels of several proteins associated with proliferation in A549 cells. These included AXL, CA9, ENO2, HER1, HER2, HER3, PRGN, and PDGF-AA (Figure 12A–H). The inclusion of BP-LCNs in the incubation decreased the expression of AXL by 42.8% (3137 ± 33.1 for control vs. 1795 ± 12.5 for BP-LCNs-treated cells, *p* < 0.001, Figure 12A), CA9 by 48.3% (2479 ± 120.2 for control vs. 1281 ± 20.5 for BP-LCNs-treated cells, *p* < 0.05, Figure 12B), ENO2 by 22.2% (8607 ± 88.2 for control vs. 6700 ± 69.3 for BP-LCNs-treated cells, *p* < 0.01, Figure 12C), HER1 by 5.9% (6455 ± 20.5 for control vs. 6077 ± 5.6 for BP-LCNs-treated cells, *p* < 0.01, Figure 12D), HER2 by 26.9% (736 ± 4.9 for control vs. 538 ± 18.4 for BP-LCNs-treated cells, *p* < 0.01, Figure 12E), HER3 by 38.8% (771 ± 36.1 for control vs. 472 ± 43.0 for BP-LCNs-treated cells, *p* < 0.05, Figure 12F), PRGN by 36.2% (7552 ± 176 for control vs. 4819 ± 306 for BP-LCNs-treated cells, *p* < 0.05, Figure 12G), and PDGF-AA by 33.6% (568 ± 35.1 for control vs. 377 ± 12.2 for BP-LCN-treated cells, *p* < 0.05, Figure 12H).

### 3.8. BP-LCNs Decreased Expression of Proteins Associated with Migration or Metastasis in A549 Cells

**Figure 13:**
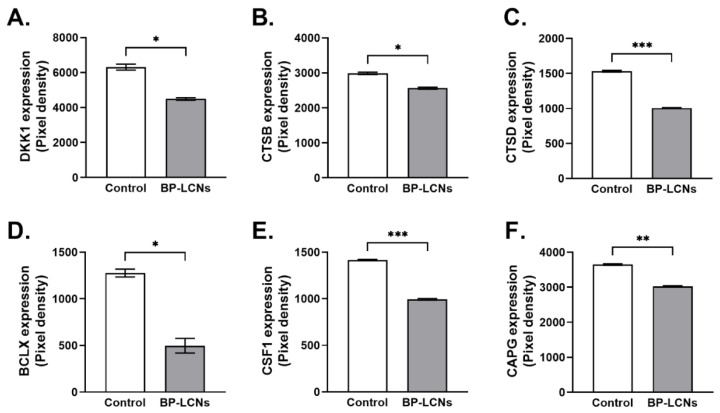
Effects of BP-LCNs on expression levels of proteins related to migration in A549 cells. A549 cells were incubated at 37 °C for 24 h in the absence or presence of BP-LCNs (final concentration 5 µM). The figure shows protein expression levels of DKK1 (**A**), CTSB (**B**), CTSD (**C**), BCLX (**D**), CSF1 (**E**), and CAPG (**F**). The protein expression levels were determined using an oncogene protein array kit and by quantifying pixel densities of the respective proteins. The values represent means ± SEM of 3 independent experiments. Note: * *p* < 0.05, ** *p* < 0.01, *** *p* < 0.001. Abbreviations: BP-LCNs, berberine–phytantriol liquid crystalline nanoparticles; DKK1, Dickkopf Wnt signaling pathway inhibitor 1; CTSB, cathepsin B; CTSD, cathepsin D; BCLX, bcl-2 like protein, CSF1, colony stimulating factor 1; and CAPG, capping actin protein.

Based on the protein profiler array data, BP-LCNs also decreased the expression of proteins associated with cell migration or metastasis in A549 cells that were incubated with BP-LCNs. These included DKK1, CTSB, CTSD, BCLX, CSF1, and CAPG proteins. The inclusion of BP-LCNs in the incubation decreased the expression of DKK1 by 28.7% (6305 ± 170 for control vs. 4494 ± 68.0 for BP-LCNs-treated cells, *p* < 0.05, Figure 13A), CTSB by 14.1% (2985 ± 33.5 for control vs. 2564 ± 29.3 for BP-LCNs-treated cells, *p* < 0.05, Figure 13B), CTSD by 32.3% (1532 ± 12.3 for control vs. 1006 ± 4.1 for BP-LCNs-treated cells, *p* < 0.001, Figure 13C), BCLX by 61.1% (1276 ± 42.5 for control vs. 497 ± 79.2 for BP-LCNs-treated cells, *p* < 0.05, Figure 13D), CSF1 by 29.8% (1415 ± 6.8 for control vs. 993 ± 8.7 for BP-LCNs-treated cells, *p* < 0.001, Figure 13E), and CAPG by 17.2% (3650 ± 22.1 for control vs. 3023 ± 17.5 for BP-LCNs-treated cells, *p* < 0.01, Figure 13F).

## 4. Discussion

This study presents evidence of the anti-cancer effects of BP-LCNs in A549 cells. The novelty of this study is that berberine hydrochloride was successfully encapsulated in phytantriol-based LCNs and optimized to achieve a novel formulation of BP-LCNs with an entrapment efficiency of 75.31 ± 0.58%. Furthermore, this study provides novel findings of significant inhibition of cell proliferation and migration and colony formation activity in A549 cells caused by incubation with the optimal BP-LCN formulation in a dose-dependent manner. The anti-cancer effects of BP-LCNs were associated with the downregulation of yet unexplored *KRT18* and upregulation of tumor suppressor genes *PTEN* and *P53* in A549 cells. In addition, the cancer marker proteins related to proliferation and metastasis or migration, such as AXL, CA9, ENO2, HER1, HER2, HER3, PRGN, PDGF-AA, DKK1, CTSB, CTSD, BCLX, CSF1, and CAPG, were significantly downregulated by BP-LCNs.

Berberine is commercially available in the solid form as berberine chloride or berberine hydrochloride, which is very slightly soluble in water [78]. Berberine, having several proven medicinal benefits, is commonly used as a supplement for the treatment of diabetes and cardiovascular diseases. However, berberine hydrochloride has poor GI absorption, with a reported absolute bioavailability of 0.68% in mice that limits its use as a therapeutic drug [35].

Our prepared BP-LCN formulations were tested for several characterization parameters (Table 1, Table 2, Table 3 and Table 4 and Figure 4, Figure 5 and Figure 6). It is a fundamental principle of experimental design that several factors and responses can be studied simultaneously using a statistical method. In return, a minimum number of studies can yield the most knowledge regarding the interactions between independent and dependent variables. In any typical formulation development process, this strategy can be used to avoid costly and time-consuming trial-and-error methods that are likely to be necessary. One of the most commonly used methods for optimizing formulation parameters is using the appropriate models. In this study, linear and quadratic models were employed for the optimization with the desired particle size and encapsulation efficiency. The pharmaceutical characterization tests showed favorable data in terms of particle size, PDI, zeta potential, entrapment efficiency, and in vitro release of BP-LCNs.

Natural compounds such as berberine, curcumin, and zerumbone share the same features of low water solubility, poor bioavailability, and low cellular uptake, thereby limiting its clinical use for a wider range of diseases [42,79]. However, with the utilization of the nanotechnology approach, it is possible to improve their physiochemical characteristics, and formulating nanoparticles could enhance their therapeutic efficacy. For example, the anti-cancer activity of curcumin nanoparticles was found to better than pure curcumin powder [42]. A previous report suggests that berberine hydrochloride inhibits the proliferation of A549 cells at concentrations higher than 90 µM, following a 24 h incubation period [26]. The findings in this study established that a greater potency of berberine can be achieved by loading berberine HCl in a phytantriol-based LCN formulation by showing significant inhibition of A549 cell proliferation, migration, and colony formation at 5 µM berberine concentration. A highly potent BP-LCN formulation, requiring a low dose for its intended effect, significantly reduced the risk of undesirable side-effects and toxic effects that are apparent with non-loaded free berberine [80,81,82].

The regulation of tumor suppressor genes *P53* and *PTEN* by BP-LCNs is consistent with the previous studies, where berberine induced anti-cancer activity in NSCLC via apoptosis, cell cycle arrest, and cellular autophagy in NSCLC and colon and ovarian cancer cells [31,33,34]. This is the first study where *KRT18* in A549 cells was found to be regulated by berberine. KRT18, a cytoskeletal protein essential for cellular integrity, is suggested to be overexpressed in most cancer types, including liver, colon, and NSCLC, and is associated with their advanced clinical stage, metastasis, malignant status, and poor prognosis and survival in patients [56,83,84]. A transient knockdown of *KRT18* decreases cell migration in lung cancer cells [56]. Taken together, the findings in this study suggest that BP-LCNs may inhibit A549 cell migration, at least in part, by downregulating *KRT18* mRNA levels.

AXL, CA9, ENO2, HER1, HER2, HER3, PRGN, and PDGF-AA are protein biomarkers of cancer that are associated with the proliferation and angiogenesis of cancer, and are potential targets for anti-cancer therapy [58,59,60,61,62,63,64,65]. On the other hand, DKK1, CTSB, CTSD, BCLX, CSF1, and CAPG are associated with the metastatic potential of cancer [66,67,68,69,70,71,72]. The downregulation of these proteins in A549 cells incubated with BP-LCNs may be directly or indirectly involved in the observed inhibition of the proliferative and migratory activity of A549 cells. These findings also suggest that BP-LCNs may provide anti-cancer therapeutic benefit by acting on multiple protein targets through multiple mechanisms.

Various in vitro experimental models are commonly used to unravel the molecular mechanism associated with the progression of chronic respiratory disease [85]. Although we observed potent in vitro anti-cancer activity of BP-LCN formulations, there are several limitations to our study that are potential areas for future research. As our study was completely in vitro using A549 cell line, new platform could possibly be used by us and other researchers to carry out in-depth studies using pre-clinical animal models (in vivo) of lung cancer. Further pharmacokinetic and anti-cancer studies of BP-LCN formulations in in vivo animal models could elucidate its potential benefits in the treatment of NSCLC and develop it as a therapeutic drug dosage form. This study also encourages further research in investigating the anti-cancer efficacy of BP-LCNs in other lung cancer cell lines, for example Calu3 and H460 cells. Overall, the utilization of nanotechnology and the drug delivery approach in formulating berberine can improve its physiochemical parameters, efficacy, stability, and cellular uptake, for its potential development as an alternative drug therapy for the management of lung cancer.

## 5. Conclusions

The findings in this study provide clear evidence of the advantage of the nanoformulation of phytantriol-based berberine over non-loaded free berberine by showing a better drug release profile and ~20 times higher potency in inhibiting the growth and metastasis of NSCLC compared to free berberine. The in vitro study provides evidence for the use of the BP-LCN formulation in the regulation of the expression of *P53*, *PTEN*, and *KRT18* genes related to cancer and the downregulation of proteins involved in cancer cell migration and proliferation. Our BP-LCN formulation can potentially address the low solubility, low oral bioavailability, low efficacy, and adverse side effects of free berberine. Thus, the phytantriol-based nano-drug delivery system incorporating berberine is a promising candidate for development as a new therapeutic drug dosage form for the treatment of lung cancer.

## Figures and Tables

**Table 1:** Compositions of BP-LCN formulations and their characterization parameters. The PS and EE values are represented as means ± SD (n = 3).

Formulation	Conc. of Phytantriol (% *w*/*w*)	Conc. of P407 (% *w*/*w*)	Berberine Hydrochloride (% *w*/*w*)	Water	Sonication Amplitude (%)	PS (nm)	EE (%)
BP-LCN1	1	10	0.1	Up to 5 mL	20	288 ± 1.8	62 ± 0.6
BP-LCN2	2	10	0.1	Up to 5 mL	20	460 ± 2.0	75 ± 0.7
BP-LCN3	4	10	0.1	Up to 5 mL	20	566 ± 2.5	75 ± 0.7
BP-LCN4	1	10	0.1	Up to 5 mL	40	185 ± 1.2	74 ± 0.7
BP-LCN5	2	10	0.1	Up to 5 mL	40	228 ± 1.7	75 ± 0.7
BP-LCN6	4	10	0.1	Up to 5 mL	40	324 ± 2.0	84 ± 0.9
BP-LCN7	1	10	0.1	Up to 5 mL	80	247 ± 1.8	76 ± 0.7
BP-LCN8	2	10	0.1	Up to 5 mL	80	209 ± 1.7	74 ± 0.7
BP-LCN9	4	10	0.1	Up to 5 mL	80	369 ± 2.0	84 ± 0.9

Abbreviations: P407, Poloxamer 407; PS, particle size; EE, entrapment efficiency.

**Table 2:** ANOVA for the quadratic model of particle sizes of BP-LCNs.

Source	Sum of Squares	Df	Mean Square	F-Value	*p*-Value	
Model	1.420 × 105	5	28,409.95	18.34	0.0007	Significant
A—Conc. of lipid	46,542.57	1	46,542.57	30.04	0.0009	Significant
B—Sonication amplitude	40,264.63	1	40,264.63	25.99	0.0014	Significant
AB	2365.18	1	2365.18	1.53	0.2565	Not significant
A²	0.0057	1	0.0057	3.697 × 10−6	0.9985	Not significant
B²	49,193.02	1	49,193.02	31.75	0.0008	Significant
Residual	10,845.03	7	1549.29			
Lack of Fit	10,845.03	3	3615.01			
Pure Error	0.0000	4	0.0000			
Cor Total	1.529 × 105	12				

**Table 3:** ANOVA for the linear model of the entrapment efficiency of BP-LCN formulations.

Source	Sum of Squares	Df	Mean Square	F-Value	*p*-Value	
Model	216.72	2	108.36	9.39	0.0051	Significant
A—Conc. of lipid	158.94	1	158.94	13.78	0.0040	Significant
B—Sonication amplitude	53.71	1	53.71	4.66	0.0563	Significant
Residual	115.37	10	11.54			
Lack of Fit	115.37	6	19.23			
Pure Error	0.0000	4	0.0000			
Cor Total	332.09	12				

**Table 4:** Characterization parameters of BP-LCNs.

Parameters	BP-LCNs
Z-average (nm)	223 ± 1.8
Polydispersity Index (PdI)	0.34 ± 0.01
Zeta potential (mV)	−15.7 ± 0.1
Encapsulation efficiency (%)	75 ± 0.6

## Data Availability

The data presented in this study are available on request from the corresponding author.
